# Supplementary material for: All-at-once RNA folding with 3D motif prediction framed by evolutionary information
Source: Nat Methods. 2025 Oct 3;22(10):2094–106. doi: 10.1038/s41592-025-02833-w (PMC12510878; doi:10.1038/s41592-025-02833-w)
Supplement: Supplementary file 3 — Source code to run CaCoFold-R3D. Data and results. [file 41592_2025_2833_MOESM3_ESM.gz › supplemental_material/results/CaCoFold-R3D/CaCoFold-R3D_Rfam/RF02147_MEG8_3.R2R.sto.pdf]

RF02147_MEG8_3

5′-UUGGCUUGUUCAARUCAGUGUYCAAAYUUYUCCUGCAUUUCARGGGGCCUGGGRUUYRGRAG
